# Advising Obese Adults about Diet and Physical Activity in Sousse, Tunisia

**DOI:** 10.1155/2013/498527

**Published:** 2013-02-21

**Authors:** Jihene Maatoug, Imed Harrabi, Sonia Hmad, Mylene Belkacem, Asma Nouira, Hassen Ghannem

**Affiliations:** Department of Epidemiology, University Hospital Farhat Hached, Sousse, Tunisia

## Abstract

*Background*. To our knowledge no study has been conducted in Tunisia to describe practice of health care providers towards chronic disease risk factors, particularly among obese adults. *Aim*. This study is aimed at assessing the level of giving advice on diet and physical activity by health care providers for obese adults comparing to nonobese adults in Tunisia. *Methods*. A cross-sectional survey was carried out in 2010 for adults aged from18 to 65 years living in the region of Sousse. The questionnaires were administered by an interview made by trained data collectors to standardize the administration of the questionnaire. Anthropometric measurements of height and weight were obtained using a standardized protocol from each participant. *Results*. The findings from this study indicate that obesity is frequent among adults essentially among women and aged categories. It also shows that obese adults are not adequately diagnosed with few proportion of anthropometric measure evaluation among all the participants. Even if obese participants were significantly more advised than nonobese participants with, respectively, 28.5% to lose weight and 23.8% to increase physical activity, this proportion remains low.

## 1. Introduction 

Obesity has become a growing global health problem. There are approximately 937 million and 396 million obese and overweight adults worldwide, respectively [1]. Southern and eastern Mediterranean countries have been particularly affected by this evolution [2, 3]. However, the obesity has well-known associations with all-cause mortality [4, 5], morbidity [6], and disability, resulting in unhealthy life years with poor quality of life [7, 8] and increased health care costs [9, 10].

The World Health Organization report on obesity states that sedentary lifestyle and consumption of high-fat energy-dense diets are fundamental causes of the obesity epidemic [11]. Health promotion strategies, including behavioral interventions aimed at modifying dietary habits and physical activity patterns, are essential in prevention and management of obesity. A substantial part (60–70%) of population makes visits to their general practitioner (GP) each year [12]. However, the studies have shown low rate of counseling on lifestyle changes given to overweight patients in primary health care [13, 14].

Health care providers such as general practitioner, and nurses have the opportunity to confront this epidemic and to educate their patients about weight loss. Physicians and other health care professionals may, however, be missing an important opportunity to counsel overweight individuals to lose weight or to maintain their weight and prevent morbidity and mortality [15].

In recent years, there has been much research-based focus on using the general practice setting for health promotion including improving dietary and exercise practices among patients, particularly for weight management. To our knowledge no study has been conducted in Tunisia to describe practice of health care providers towards chronic disease risk factors, particularly among obese adults.

This study is aimed at assessing the level of giving advice on diet and physical activity by health care providers for obese and nonobese adults in Tunisia.

## 2. Material and Methods

### 2.1. Study Design

We conducted a cross-sectional survey carried out in 2010. The target population was all adults aged from 18 to 65 years. 

### 2.2. Study Population

The sampling frame was derived by the Tunisian National Institute of Statistics from the database of the most recent census of the population carried out in 2004. According to this list of households, we used a random representative sample of the general urban population of Sousse Jawhara, Sousse Riadh, and Msaken by selecting districts from these regions. Then, all adults aged from 18 to 65 years living in these districts and who were present at data collection were included.

### 2.3. Data Collection

We used an Arab pretested questionnaire to collect data about health status, chronic disease risk factors, and care received in medical consultation for participants. The questionnaires were administered by interview by trained data collectors to standardize the administration of the questionnaire.

Anthropometric measurements of height and weight were obtained using a standardized protocol from each participant during interview and clinical examination. Height was measured, to the nearest 0.5 cm, without shoes, with the participant's back square against the wall tape, eyes looking straight ahead with a right-angle triangle resting on the scalp and against the wall. Weight was measured with a lever balance to the nearest 100 grams, without shoes, in light undergarments. Body mass index (BMI) was calculated as weight (kg) divided by the square of the height (m) and obesity was defined as BMI > 30 kg/m_2_ [16].

### 2.4. Statistical Analysis

The mean ± SD values were calculated for continuous variables and the % age of each subgroup for categorical variables. Comparisons between two group means were performed by using the *t-*test. Associations between categorical variables were tested by using contingency tables and the *χ* ^2^ test. All data analyses were conducted using SPSS statistical software (Version 10.0). All statistical tests were 2-tailed, and *P* values < 0.05 were considered statistically significant.

### 2.5. Ethical Consideration

The study was approved by the Ethics Committee of the University Hospital Farhat Hached, Tunisia. It does not represent any risk for participants who gave their consent before responding to the questionnaire.

## 3. Results

The response rate was 72.1%. Our population was composed of 1880 adult participants with 64% of women. The mean age of participants was 37.9 ± 13.5 years. The prevalence of obesity was 18.1% and 33.5%, respectively, among men and women. 

The prevalence of obesity increased significantly with age in both sex (Table 1).

Demographic characteristics of the study population by obesity status are provided in Table 2. The obese participants were more likely to be married, did not work, and had a level of education less than 6th grade. In fact, only 9.7% of obese participants were single versus 40.8 single persons among nonobeses.

Only 6 obese participants (1.2%) were diagnosed as having obesity by a health professional as reported by participants.

Among obese participants, 42.3% reported feeling good health status versus 64.3% among nonobese participants (*P* < 10^−3^). However, there was not a significant difference between them concerning feeling stressed or under a lot of pressure, or depressed or bothered by little interest or pleasure in doing things (Table 3). Concerning chronic diseases risk factors, obese participants were more likely to have high blood pressure, hypercholesterolemia, and diabetes in their personal antecedents (Table 3). 

Among obese participants, 178 (70.1%) were screened as having high blood pressure but they were not diagnosed. This proportion was 82.9% (281 participants) among nonobese participants. 

The proportion of obese adults who had visited a physician in the previous twelve months was 74.3% versus 64.8% among nonobese participants with significant difference (*P* < 10^−3^).

Among obese adults who had visited a physician in the previous twelve months, an estimated 30.9% reported that their weight was taken by a health care provider versus 25.5% among nonobese adults (*P* = 0.05) (Table 4). Hip and waist circumference were taken at almost the same proportion for obese and nonobese consultants with, respectively, 5.5% and 7.1% among obese and 5.2% and 4.8% among nonobese (Table 4). Blood pressure was taken in 67.4% and 50.1% of cases respectively for obese and nonobese adults (*P* < 10^−3^). Respectively, blood cholesterol and blood glucose were taken significantly more frequently among obese consultants. 

The proportion of obese adults who had visited a physician in the previous twelve months reported that a health care provider discussed with them their weight status in 30.4% of cases and being advised to lose weight in 28.5% of cases (Table 5). They received advice about how to follow a healthy diet in 28.6% versus 15.7% for nonobese participants with significant difference. Health care providers gave advice or treatment to increase physical activity in 23.8% and 11.7%, respectively, for obese and nonobese participants. However, they discussed with their patients their risk for diabetes, cardiovascular disease (CVD), and/or cancer only in almost 4% of cases for the two groups (Table 5).

## 4. Discussion

The findings from this study indicate that obesity is frequent among adults essentially among women and aged categories. It also shows that obese adults are not adequately diagnosed with few proportion of anthropometric measure evaluation among all the participants.

Even if obese participants were significantly more advised than nonobese participants, this proportion remains low. Other studies that evaluated counseling obese patients by GPs have shown that proportion of advised persons varies from 5% to more than 40% [13, 14]. In the USA [17] 39% of obese adults who had visited their GP in the previous 12 months were advised to lose weight. In another study, 34% reported being counseled about exercise at their last visit and patients who were overweight to obese were more likely to be counseled [18].

Sciamanna et al. [15] revealed that advice to lose weight is uncommon and is given primarily to those who are already obese. Only about 17% in this study, individuals who have visited a physician and who have health care coverage indicated receiving physician's advice to lose weight even though 38% of these individuals are overweight and 24% are obese.

The rates of obesity and overweight in our study were similar to a nation-wide representative sample of Tunisian [19]. GPs may not offer lifestyle advice as they view it as ineffective, although GPs appear to agree that nutrition is important in managing disease [20]. GPs often face many barriers to offer lifestyle advice to patients, particularly if the presenting problem is unrelated. GPs may lack confidence in offering more detailed nutrition advice [21] due, in part, to insufficient knowledge [22]. Several studies have shown that GPs knowledge about nutrition and physical activity in management of obesity is incomplete [23]. They expressed the need for clinical guidelines and supplementary training. Even when GPs have nutritional knowledge, they find it difficult to communicate this knowledge effectively. GPs would therefore benefit from additional training in counseling skills.

Other barriers include time constraints, a lack of incentives or reimbursements [24], complexity of advice, lack of training in counseling skills, a lack of interest [25], the idea that patients are not motivated [21], and a long delay between intervention and observable effects [26].

Diet and physical activity are relatively time consuming to assess; there is no simple measurement tool, and results are open to misinterpretation by patients. The authors found that more GPs reported usually “offering advice” than “assessing readiness to change,” which suggests that patients may have been receiving advice that they were not ready to listen to or act upon. Routine assessment of readiness to change before offering suggestions or advice may yield better results for lifestyle counseling [27].

Therefore, a discrepancy seems to exist between the recommendations patients report to have received from their healthcare professionals and clinical guidelines, as well as findings from research studies on the prevalence and content of weight loss advice [28].

Whatever the exact reasons for the low propensity to give advice, it is imperative that physicians and other health care professionals are encouraged through various programs or approaches (training about obesity, intervention strategies, public policy, etc.) to do more to advice their overweight and obese patients about weight loss [29].

## 5. Conclusion 

Chronic disease risk factors are common in the Tunisian population, and health care providers are ideally placed to offer assessment, advice, and referral to services and programs. Future studies that would determine the predictive factors of counseling patients to lose weight and directly measure the effect of physician's advice on actual weight or behavior over time are needed to address this issue further.

## Figures and Tables

**Table 1:** Prevalence of the obesity by sex and age among adults aged from 18 to 65 years in Sousse.

	Men n (%)	Women n (%)	*P*
Obesity (BMI > 30 kg/m^2^)	111 (18.1)	395 (33.5)	**<0.001 **
Age			
<30 years	20 (7.2)	48 (12.0)	
30–45 years	35 (20.8)	149 (37.3)	**<0.001**
>45 years	64 (31.1)	196 (52.1)	

**Table 2:** Sociodemographic distribution of adults according to obesity status in the region of Sousse, Tunisia.

	Obesity		
	No *n* (%)	Yes *n* (%)	*P*
Marital status (single)	538 (40.8)	50 (**9.7**)	**<0.001**
Level of education less than 6th grade	365 (27.6)	260 (50.7)	**<0.001**
Do not work	189 (63.0)	314 (61.3)	**<0.001**

**Table 3:** Health status and antecedents of adults according to obesity status in the region of Sousse, Tunisia.

	Obesity		
	No *n* (%)	Yes *n* (%)	*P*
Feel* good health *status	694 (64.3)	187 (42.3)	**<0.001**
Feel* stressed *or under a lot of pressure	286 (21.6)	131 (25.5)	0.07
Feel depressed	549 (41.6)	209 (40.7)	0.71
bothered by* little interest *or pleasure in doing things	528 (40.0)	199 (38.7)	0.62
Have diagnosis of *high blood pressure *	69 (5.2)	97 (18.9)	**<0.001**
Have diagnosis of* high cholesterol *	26 (2.0)	44 (8.6)	**<0.001**
Have diagnoses of* diabetes *	50 (3.8)	62 (12.1)	**<0.001**

**Table 4:** Distribution of tests and/or measurements taken by a health care provider at any time in the past 12 months among adults by obesity status in the region of Sousse, Tunisia.

	Obesity		
	No *n* (%)	Yes *n* (%)	*P*
Have you* visited *a health care facility in the past 12 months for your routine health care	856 (64.8)	382 (74.3)	**<0.001**
*Weight *was taken by a health care provider at any time in the past 12 months	217 (25.5)	117 (30.9)	0.05
*Height *was taken by a health care provider at any time in the past 12 months	138 (16.2)	74 (19.5)	0.15
*Hip circumference *was taken by a health care provider at any time in the past 12 months	44 (5.2)	21 (5.5)	0.79
*Waist circumference *was taken by a health care provider at any time in the past 12 months	41 (4.8)	27 (7.1)	0.11
*Blood cholesterol *was taken by a health care provider at any time in the past 12 months	203 (23.9)	169 (44.7)	**<0.001**
*Blood pressure *was taken by a health care provider at any time in the past 12 months	427 (50.1)	256 (67.4)	**<0.001**
*Blood glucose *was taken by a health care provider at any time in the past 12 months	240 (28.2)	193 (51.1)	**<0.001**

**Table 5:** Distribution of advices, and treatments given by a health care provider at any time in the past 12 months among adults by obesity status in Sousse, Tunisia.

	Obesity		
	No *n* (%)	Yes *n* (%)	*P*
Discuss with you your current weight status?	94 (11.0)	116 (30.4)	**<0.001**
Give you advice or treatment to lose weight?	72 (8.4)	109 (28.5)	**<0.001**
Discuss with you your current diet?	139 (16.3)	106 (27.7)	**<0.001**
Give you advice about how to follow a healthy diet?	134 (15.7)	109 (28.6)	**<0.001**
Discuss with you your current level of physical activity?	107 (12.5)	95 (24.9)	**<0.001**
Give you advice or treatment to increase physical activity?	100 (11.7)	91 (23.8)	**<0.001**
Discuss with you your risk for diabetes, cardiovascular disease (CVD), and/or cancer?	39 (4.6)	18 (4.7)	0.92
